# Genetic association study of NLRP1, CARD, and CASP1 inflammasome genes with chronic Chagas cardiomyopathy among *Trypanosoma cruzi* seropositive patients in Bolivia

**DOI:** 10.1371/journal.pone.0192378

**Published:** 2018-02-13

**Authors:** Steven J. Clipman, Josephine Henderson-Frost, Katherine Y. Fu, Caryn Bern, Jorge Flores, Robert H. Gilman

**Affiliations:** 1 Department of International Health, Johns Hopkins Bloomberg School of Public Health, Baltimore, Maryland, United States of America; 2 Massachusetts General Hospital, Boston, Massachusetts, United States of America; 3 University of Pittsburgh Medical Center, Pittsburgh, Pennsylvania, United States of America; 4 Department of Epidemiology and Biostatistics, University of California San Francisco School of Medicine, San Francisco, California, United States of America; 5 Hospital San Juan de Dios, Santa Cruz de la Sierra, Bolivia; 6 Catholic University of Bolivia, Santa Cruz, Bolivia; University of the Pacific, UNITED STATES

## Abstract

About 20–30% of people infected with Chagas disease present with chronic Chagas cardiomyopathy (CCC), the most serious and frequent manifestation of the disease, while others remain asymptomatic and often do not experience Chagas-specific mortality. It is not currently well understood what causes these differential disease outcomes, but a genetic predisposition within the host could play an important role. This study examined variants in the NLRP1, CARD, and CASP1 inflammasome genes among 62 *T*. *cruzi* seropositive patients from Bolivia (38 cases with CCC and 24 asymptomatic controls) to uncover associations with CCC. All subjects underwent a complete medical examination including electrocardiogram (EKG) and echocardiogram. After genotype calling and quality control filtering with exclusion of 3 cases and 3 controls, association analysis was performed across 76 directly genotyped SNPs in NLRP1, CARD, and CASP1 genes, adjusting for age, sex, and population stratification. One SNP (rs11651270; Bonferroni-corrected p = 0.036) corresponding to a missense mutation in NLPR1 was found to be significant after adjustment for multiple testing, and a suggestive association was seen in CARD11 (rs6953573; Bonferroni-corrected p = 0.060). Although limited by sample size, the study results suggest variations in the inflammasome, particularly in NLRP1 and CARD11, may be associated with CCC.

## Introduction

American trypanosomiasis, or Chagas disease as it is more commonly known, is a neglected tropical disease caused by the protozoan parasite *Trypanosoma cruzi (T*. *cruzi)*. Largely endemic to areas of Latin America, Chagas disease leads to more morbidity and mortality in the Americas than any other parasitic disease [1, 2]. It is currently estimated that 8 million people are infected worldwide with over 70 million at risk [2, 3]. Bolivia has the highest *T*. *cruzi* infection prevalence in the world [4]. Chagas disease transmission is primarily vector-borne through several species of hematophagous triatomine insects, but can also be spread through blood transfusions, organ transplants, and congenitally [2].

Chagas disease typically presents in two distinct phases: an acute phase and a chronic phase. The acute phase, which lasts for the first few weeks or months of infection, is often asymptomatic or causes mild, non-specific symptoms [5]. Although infection persists, acute symptoms usually resolve without treatment [2]. During the chronic stage of Chagas disease, the *T*. *cruzi* parasites reside primarily in the cardiac tissues [5, 6]. The chronic stage of Chagas can range from asymptomatic (indeterminate form) to severe illness and premature death, generally occurring 20 to 40 years after acute infection [2, 7]. The clinical manifestations in this stage generally include cardiomyopathy, heart failure, cardiac arrhythmias, and cardiac arrest [2, 5, 6]. Chronic Chagas cardiomyopathy (CCC) is the most serious and frequent manifestation of Chagas disease and develops in around 20–30% of individuals, while many others remain asymptomatic and do not experience Chagas-specific mortality [6]. It is not currently well understood why less than one-third of infected individuals develop CCC. Although treatment in the chronic phase remains controversial, earlier detection of at-risk individuals is likely to allow for antiparasitic or cardiac-specific treatment to prevent disease progression [8].

Antiparasitic medications with activity against *T*. *cruzi*, such as benznidazole and nifurtimox can be given as treatment to kill the parasite. Both medications have been shown to be highly effective in the acute phase; however, efficacy has been difficult to demonstrate in the chronic phase [5, 9]. Due to the long durations of treatment (60 to 90 days), the high cost of treatment for resource poor regions, and the possibility of severe adverse reactions (occurring in up to 40% of patients) the potential benefits of preventing or delaying disease progression must weighed [5, 6]. If treatment can be targeted to those most likely to benefit from it, resources can be maximized and side-effects can be mitigated since the health and survival of the 70–80% who remain asymptomatic will not be changed by treatment.

Reliable genetic markers to identify those at risk of cardiac disease progression would allow for scarce resources in countries like Bolivia to be used to maximum effect while limiting unnecessary exposure and possible adverse effects from current drugs. Current recommendations advise discussing the balance of treatment risks and benefits with patients to inform individual decision-making [10–12]. Bearing this in mind, the use of genetic variants identified through an association study could inform better counseling of infected adults based on a more accurate assessment of risk rather than absolute recommendations for or against treatment. This research aims to advance the understanding of Chagas disease and the genetic risk factors for CCC.

Although there are limited genetic studies on CCC, there is some evidence indicating that T-cell mediated inflammation and autoimmunity contribute to inflammatory damage of myocardial tissue and conduction systems [13, 14]. The research presented in this paper utilized a subsample of participants in a cross-sectional study of CCC to characterize the genetic substructure of our population and examine CCC for associations with variants related to myocardial inflammation. We focused this association analysis specifically on genes related to the caspase-1 inflammation cascade. Caspase-1 belongs to a family of nine cysteine proteases, and plays a unique role in the innate inflammatory response and autoinflammatory diseases [15–17]. Mutations in the genes involved in the caspase-1 inflammasome have also been associated with several autoimmune and inflammatory diseases [18, 19]. Although our understanding of the inflammasome with regard to Chagas disease is still limited, variants in these regions could play an important role in CCC development among those infected with Chagas disease. We chose to focus our analysis primarily on the CASP1, CARD, and NLRP1 genes. Inflammasomes are multimeric protein complexes generally organized with inflammasome sensor molecules connecting to caspase-1 via the adaptor apoptosis-associated speck-like protein containing CARD (ASC), an adaptor protein common to all inflammasomes [20]. The ASC protein consists of two death-fold domains, one pyrin domain and one caspase activation and recruitment domain (CARD) [20, 21]. Most inflammasomes contain a nucleotide-binding oligomerization domain-like (NOD-like) receptor sensor molecule, namely NLRP1 (NOD-, LRR- and pyrin domain-containing 1) which can trigger the formation of the inflammasome. More specifically, NLRP1 can activate caspase-1 through CARD without recruiting ASC [20]. We therefore chose to examine CASP1 as well as NLRP1 and CARD, since these genes are central to the formation of the caspase-1 inflammasome and execution-phase of cell apoptosis.

## Methods

### Study design

This study was designed as a nested case-control study within a larger cross-sectional study of Chagas cardiomyopathy patients enrolled from hospital-based settings in Bolivia. Confirmed *T*. *cruzi* infection was based on having positive results on 2 different commercial assays for anti-*T*. *cruzi* IgG antibodies (Wiener Recombinant 3.0 enzyme linked immunosorbent assay, Rosario, Argentina and Chagas Polychaco indirect hemagglutination test kit, Lemos Laboratories, Santiago del Estero, Argentina). As of November 2016, a total of 3,269 males and females ≥ 18 years of age have been enrolled in the study (1,866 with *T*. *cruzi* infection). Individuals were excluded from the study if they were pregnant, unable to undergo venipuncture, or unable to provide informed consent. Participants were recruited from Hospital San Juan de Dios (HSJDD), which is a large urban hospital serving low-income populations of the city of Santa Cruz and surrounding areas. Although vector-borne *T*. *cruzi* transmission is unlikely to occur in the city, the population includes many migrants from rural areas who acquired Chagas disease earlier in life. Specific methodology, screening, and follow-up as it relates to the overarching study design is detailed in our previous publications from the study [22–26].

### Sample and marker selection

A total of 38 *T*. *cruzi*-infected cases (male n = 19, mean/median age: 62/65; female n = 19, mean/median age: 55/58) with a clinical cardiomyopathy diagnosis and 24 asymptomatic *T*. *cruzi*-infected controls (male n = 12, mean/median age: 56/56; female n = 12, mean/median age: 53/55) were selected as a stratified sample from the parent study, frequency-matched by age and sex. Patients ranged in age from 40 to 81 years. Cardiomyopathy was defined based on characteristic abnormalities on electrocardiogram (ECG) (atrial fibrillation or flutter, atrioventricular blocks, bradycardia <50 beats per minute, bundle branch blocks, junctional rhythms), and/or cardiac insufficiency based on ejection fraction (EF) by echocardiogram (EF<53% for women, <51% for men). Directly genotyped markers that fell within the CASP1, NLRP1, and CARD genes were compiled for analysis, resulting in a total of 76 SNPs to be tested (see S1 Table).

### Sample preparation

DNA was extracted from 500 μL of whole blood in 500 μL RNAlater (Thermo Fisher Scientific, Waltham, MA) that had been frozen at -20°C. Samples were centrifuged at 13,500 RPM for 2 minutes and RNAlater was decanted off. DNA extractions were done using the QIAGEN FlexiGene DNA Kit (QIAGEN, Hilden, Germany) and quantified using fluorometric quantitation through the Promega QuantiFluor dsDNA System (Promega, Madison, WI) and BioTek Synergy spectrometer (BioTek, Winooski, VT). Quality was assessed through electrophoresis on a 1% agarose gel. Extraction via phenol-chloroform, QIAGEN DNeasy Kit (QIAGEN, Hilden, Germany), and Roche DNA Isolation Kit for Mammalian Blood (Roche, Basel, Switzerland) were also tested; however, the FlexiGene DNA Kit yielded the best results for our samples, in terms of both quantity of quality. DNA was concentrated as needed to meet a minimum concentration of 50 ng/μL.

### Genotyping, SNP calling, and quality control

Samples were sent to The Broad Institute of MIT and Harvard for genotyping using the Infinium OmniExpress + Exome array (Illumina, San Diego, CA) which contains 958,497 markers. Genotype calling was performed using Birdsuite, and marker and sample quality control (QC) was performed in PLINK 1.9 (cog-genomics.org/plink) [26]. Markers that exhibited a call rate < 0.97, minor allele frequency < 0.05, and violation of Hardy-Weinberg equilibrium p-value > 0.001 were removed. Further QC examined samples for individuals with < 98% genotyping coverage, kinship with Identity By Descent (IBD) proportion < 0.5, and samples with discordant sex information. After final QC 475,514 markers and all 62 samples remained in the total QC dataset.

**Fig 1:**
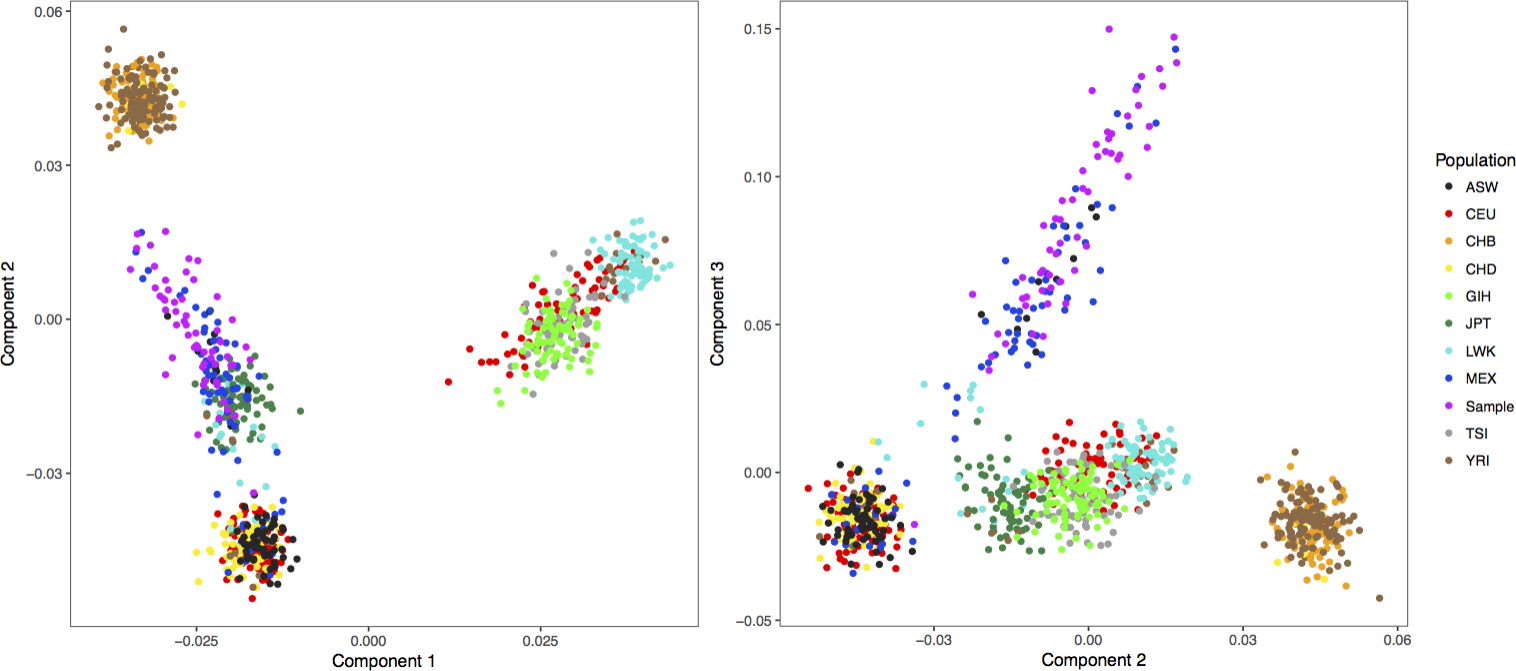
Principle component analysis of bolivian chagas samples along with HapMap 3 populations. Each point represents one individual with different colors representing each population. Bolivian Chagas samples are coded in magenta. The HapMap populations include: ASW: African ancestry in Southwest USA; CEU: Utah residents with Northern and Western European ancestry from the CEPH collection; CHB: Han Chinese in Beijing, China; CHD: Chinese in Metropolitan Denver, Colorado; GIH: Gujarati Indians in Houston, Texas; JPT: Japanese in Tokyo, Japan; LWK: Luhya in Webuye, Kenya; MEX: Mexican ancestry in Los Angeles, California; MKK: Maasai in Kinyawa, Kenya; TSI: Tuscans in Italy; YRI: Yoruba in Ibadan, Nigeria.

In order to assess population stratification and adjust for confounding through genetic admixture, individual ancestry proportions were estimated using EIGENSTRAT smartpca [27, 28]. Genome-wide ancestry informative markers were selected from the QC dataset through linkage disequilibrium (LD) pruning. The—indep command in PLINK was used to obtain a set of independent markers with an R^2^ < 0.01. The same markers were extracted from HapMap 3 samples and combined with the study data for PCA analysis. The principle components from EIGENSTRAT were visualized in R with the HapMap 3 data (Fig 1). EIGENSTRAT smartpca was also used to run a principle components analysis on the study samples alone. The within-sample components were plotted against one another in R to identify population outliers. After removing sample outliers, new principle components and eigen values were calculated with EIGENSTRAT. Visual inspection of a scree plot of the eigen values showed that the first four principle components captured the majority of the variability in admixture.

### Ethics statement

The Johns Hopkins Bloomberg School of Public Health Institutional Review Board (IRB) approved this study (entitled “Predictors of Cardiomyopathy Progression in a Chagas Disease Cohort in Bolivia”, IRB No: 00005598) on September 30, 2015 in accordance with the ethical standards laid down in the 1964 Declaration of Helsinki. The local Bolivian IRB at Universidad Católica Boliviana also approved the study (IRB No: FWA00017928) on February 3, 2016. All subjects provided written informed consent for Chagas disease screening, participation in the study, and storage of biospecimens for future research. Sample collection, storage, and SNP genotyping, was approved through the Johns Hopkins IRB, and all samples and accompanying data were fully de-identified.

## Results & discussion

As depicted in Fig 1, our samples exhibit a large degree of population substructure, closely following that of Mexican HapMap samples. It can be inferred that much of the spread stems from a similar admixed ethnic composition of indigenous groups and European subpopulations that both Mexico and Bolivia share. Examination of the within-sample principle components identified six samples as population outliers that significantly differed from the general ancestry composition of the other samples. These samples were removed to limit possible confounding, and the final sample size was reduced to 35 cases and 21 controls, comparable in age and sex.

Association analysis was run under an additive model across 76 SNPs in the NLRP1, CARD, and CASP1 genes using multivariate unconditional logistic regressions adjusting for age, sex, and genetic admixture using the first four within-sample components calculated from EIGENSTRAT smartpca. This yielded 16 SNPs with significant unadjusted p-values. Of the 76 SNPs tested, 7 markers were calculated to be LD independent. Using the number of independent markers (R^2^ < 0.03) for *m* in the Bonferroni equation, unadjusted p-values below p = 0.00714 were considered significant after adjustment for multiple testing (see Table 1).

**Table 1:** Suggestive association results of SNPs in NLRP, CARD, CASP1 genes under an additive model unconditional logistic regression adjusted for population substructure, age, and sex. P-values reflect Bonferroni adjustment for multiple testing.

SNP	Chromosome	Position	P-value (adjusted)	Gene
rs11651270	17	5425077	0.036	NLRP1
rs6953573	7	3081727	0.060	CARD11
rs9303193	17	5432405	0.069	NLRP1
rs2301582	17	5436263	0.069	NLRP1
rs11982651	7	2942273	0.087	CARD11
rs1621828	7	2944263	0.158	CARD11
rs6461749	7	3032284	0.164	CARD11
rs12417050	11	104936010	0.177	CASP1

Association analysis resulted in one significant SNP (rs11651270, Bonferroni-corrected p = 0.036), which corresponds to a missense mutation in NLRP1. This particular SNP has been associated with susceptibility and outcomes in variety of diseases where manifestations or susceptibility may be driven by variable activation of immune system response, such as HPV, bacterial meningitis, melanoma, and Crohn’s disease [29–32]. Members of the NLRP gene family are thought to be important regulators of immune response. The gene product interacts with components of the IkB kinase (IKK) enzyme complex which is involved in propagating the inflammatory response, and can regulate both caspase-1 and NF-kB (nuclear factor kappa-light-chain-enhancer of activated B cells) activity, and are key mediators in programmed cell death [33]. In addition to the association seen in NLRP1, a suggestive association was seen in CARD11 (rs6953573; Bonferroni-corrected p = 0.060). CARD11, or Caspase Recruitment Domain Family Member 11, is involved in the recruitment and activation of the inflammasome and interacts with BCL10, a protein known to function as a positive regulator of cell apoptosis and NF-kappaB activation. Mutations in CARD11 have been associated Severe Combined Immunodeficiency 11 (MCID: IMM062, malacards.com)[34] and with B-Cell Expansion with Nf-Kb and T-Cell Anergy Disease (MCID: BCL010, malacards.com)[34], which presents with symptoms of splenomegaly, recurrent infections and IgM deficiency.

There are limited studies specifically examining seropositive CCC patients versus indeterminate seropositive carriers for causal variants associated with cardiomyopathy development. To our knowledge the only genome-wide association study (GWAS) on Chagas disease to date has been in Brazil by Xutao Deng and colleagues [35]. The research focused on CCC candidate gene association studies in Brazil through a retrospective cohort study of seropositive blood donors in the National Heart, Lung and Blood Institute [35]. Their data showed a wide range of African, European, and Native American admixture characteristic of Brazil, and failed to uncover any SNPs associated with CCC at genome-wide significance.

Our study analyzed 76 SNPs in the NLRP1, CARD, and CASP1 genes for an association with cardiomyopathy among seropositive Chagas disease patients with and without CCC. We identified a significant association at rs11651270 in NLRP1 and a suggestive association at rs6953573 in CARD11, implying that NLRP1 and CARD11 are associated with CCC. The study is limited in power by its small sample size; however, this work demonstrates that mutations in the inflammasome display an association with CCC and serves as a starting point to guide future studies in the field. Further research is needed to see if the observed association plays a causal role in myocardial inflammation. A recent study showed that NLRP1 has also been associated with peripheral artery disease, making it an interesting target for future research [36].

Overall, this study begins to characterize the population substructure of our Bolivian samples and identifies targets possibly associated with CCC that can be further examined in a large-scale GWAS. Identification of genetic variants associated with CCC has the potential to allow for tailored treatment via early detection of individuals at risk for cardiomyopathy. Moreover, validating and extending this study to identify variants at genome-wide significance is of utmost importance for understanding the disease pathophysiology and host genetic factors contributing to CCC.

## Supporting information

S1 Dataset PED and MAP files of raw minimal anonymized dataset. (ZIP)

S1 Table List of SNPs tested in association analysis of NLRP1, CARD, and CASP1 genes with CCC. (DOCX)
