# Supplementary material for: Genetic association study of NLRP1, CARD, and CASP1 inflammasome genes with chronic Chagas cardiomyopathy among Trypanosoma cruzi seropositive patients in Bolivia
Source: PLoS One. 2018 Feb 13;13(2):e0192378. doi: 10.1371/journal.pone.0192378 (PMC5810984; doi:10.1371/journal.pone.0192378)
Supplement: S1 Table — (DOCX) [file pone.0192378.s002.docx]

**Supporting Information**

**S1 Table. List of SNPs tested in association analysis of NLRP1, CARD, and CASP1 genes with CCC.**

| SNP | | |
| --- | --- | --- |
| rs12417050 | rs4610634 | rs1792753 |
| rs6953573 | rs2396971 | rs2029279 |
| rs12797863 | rs1843939 | rs17178663 |
| rs10951010 | rs958331 | rs12671372 |
| rs11651270 | rs2679261 | rs10951005 |
| rs10951014 | rs6947877 | rs12950235 |
| rs3735124 | rs2301920 | rs2001363 |
| rs9303193 | rs4991791 | rs4590335 |
| rs2301582 | rs1182136 | rs3744717 |
| rs10951015 | rs10280353 | rs4577136 |
| rs11982651 | rs13245486 | rs4722304 |
| rs6461749 | rs11654176 | rs10236776 |
| rs11657333 | rs1713911 | rs4722332 |
| rs1843933 | rs1878806 | rs6949660 |
| rs4722366 | rs2301921 |  |
| rs9986734 | rs2527506 |  |
| rs1713910 | rs7810813 |  |
| rs4722353 | rs3735118 |  |
| rs2679258 | rs10950998 |  |
| rs1621828 | rs3735119 |  |
| rs6970813 | rs1878805 |  |
| rs9913271 | rs6461781 |  |
| rs3926687 | rs6461701 |  |
| rs11650176 | rs1636167 |  |
| rs11657249 | rs1078230 |  |
| rs11656977 | rs11982868 |  |
| rs3735120 | rs1182137 |  |
| rs3735123 | rs6461796 |  |
| rs3735126 | rs6461700 |  |
| rs1621509 | rs925592 |  |
| rs1124581 | rs11769046 |  |
